# The Use of Micro-Credentials in Health Professions Education: A Scoping Review

**DOI:** 10.5334/pme.2038

**Published:** 2025-12-05

**Authors:** Marco Zaccagnini, Andrew J. West, Brandon D’Souza, Peter Farrell, Sébastien Tessier, Ian D. Graham

**Affiliations:** 1 Postdoctoral Fellow, School of Epidemiology and Public Health, University of Ottawa, Ottawa, ON, Canada; 2 The Canadian Society of Respiratory Therapists, Ottawa, ON, Canada; 3 Chief Executive Officer, The Canadian Society of Respiratory Therapists, Ottawa, ON, Canada; 4 Anesthesia Assistant, Department of Paediatric Anaesthesia, IWK Health Centre, Halifax, NS, Canada; 5 Research Librarian, Health Sciences Library, University of Ottawa, Ottawa, ON, Canada; 6 Registered Respiratory Therapists, Health Services – Acute Care, Winnipeg Regional Health Authority, Winnipeg, MB, Canada; 7 Professor, School of Epidemiology and Public Health, University of Ottawa, Ottawa, ON, Canada; 8 Centre for Practice-Changing Research, Ottawa Hospital Research Institute, Ottawa, ON, Canada; 9 School of Nursing, University of Ottawa, University of Ottawa, Ottawa, ON, Canada

## Abstract

**Background:**

Healthcare professionals must continuously update their competencies to keep pace with evolving clinical practices; however, traditional continuing professional development (CPD) methods often have limited impact on competency and performance. Micro-credentials have emerged as a flexible and personalized alternative to traditional CPD, yet little is known about how they are designed, implemented, and evaluated in health professions education. As educational systems invest heavily in micro-credentials, a clearer understanding of their educational value is essential.

**Methods:**

Using a scoping review guided by Arksey and O’Malley’s six-stage framework and PRISMA-ScR guidelines, we systematically searched seven databases (inception–December 2024) and conducted a structured grey literature review. We examined the instructional design features, pedagogical underpinnings, assessment strategies, and reported impacts of micro-credentials.

**Results:**

We included 19 peer-reviewed papers and 35 websites describing health-related micro-credentials. Most studies were published in 2024 (42.1%), originated from the United States (42.1%), and nearly half (47.4%) provided only descriptive accounts. A wide range of instructional design features were identified, though pedagogical theories were rarely stated. Assessment strategies predominantly emphasized summative approaches (e.g., multiple-choice knowledge checks), with limited focus on higher-level competency assessment. Reported outcomes were primarily improvements in knowledge, confidence, or engagement, with no clear evidence of the distinct value of micro-credentials as a teaching modality.

**Discussion:**

Current literature offers limited evaluation of micro-credentials and often lacks theory-informed design. We infer a pedagogical foundation aligned with constructivist, context-sensitive, and stage-based principles, which may inform the development of future micro-credential programs.

## Background

Healthcare professionals work in dynamic and evolving environments where advancements in research, new technologies, treatments, and shifting patient needs require continuous practice adaptation [1]. Providing high-quality care requires professionals to develop their competencies beyond the foundational knowledge acquired during formal education. To meet these demands, most regulated professions mandate lifelong learning through continuing professional development (CPD) to ensure professionals maintain and enhance their competencies. This ongoing expectation of lifelong learning is essential for delivering safe, effective, and evidence-informed care that meets the evolving demands of professional practice [2].

Educational and training programs designed to support healthcare professionals’ lifelong learning are categorized in various ways, including CPD, continuing medical education, or continuing education [3,4]. In this paper, we use the term CPD to refer to all educational programs aimed at maintaining and advancing professional competencies over time. These CPD programs encompass both structured activities, such as formal courses, and informal methods, like self-directed learning [3,4]. Traditional CPD methods, such as reading journals, attending conferences, and participating in workshops, have supported efforts to both maintain their existing competencies and advance their skills to keep pace with the evolving healthcare landscape by disseminating knowledge and facilitating peer learning [3,5]. However, these approaches have been found to have a limited effect on improving professionals’ competence and performance, with no significant effect on patient health outcomes [4,6,7].

Despite their widespread use, traditional methods of CPD present several practical challenges. Many clinicians face time constraints that limit their ability to engage meaningfully with CPD during working hours, often forcing them to rely on personal time despite demanding clinical workloads [7,8,9]. In-person CPD events may also be inaccessible due to time and travel costs [8,9,10]. Furthermore, the fragmented nature of CPD participation makes it difficult for healthcare professionals to systematically track their progress or integrate new learning into practice [9,11]. Without a structured framework to guide skill development and ensure continuity, CPD efforts may not lead to meaningful changes in clinical practice.

In response to these challenges, changes caused by the COVID-19 pandemic, and the rapid digitization of knowledge enabled by artificial intelligence, the development and adoption of micro-credentials have accelerated as a strategy for learners to expand their knowledge, skills and competencies in a flexible way that is targeted to personal and professional needs [12,13,14,15,16]. Micro-credentials are short, focused learning experiences, often designed to certify, reskill, or upskill specific competencies, skills, or knowledge areas and emphasize measurable learning outcomes rather than time spent learning, aligning with competency-based education principles [12,13,14,15]. They are intended to offer a personalized and flexible approach to learning, allowing healthcare professionals to acquire targeted skills that match their specific needs and goals. For example, a respiratory therapist might complete a micro-credential focused on advanced mechanical ventilator management [17], or a registered nurse might complete one focused on electrocardiogram rhythm analysis [18], each possibly addressing a knowledge gap identified through self-reflection or by an employer. Micro-credentials are purported to enable busy clinicians to continue learning while providing verified documentation of their progress. They may complement existing qualifications or function as stand-alone units, addressing emerging areas of practice or gaps in professionals’ knowledge or skills. This flexibility makes them particularly suitable for healthcare professionals, who need accessible, adaptable, and relevant learning opportunities to stay current in a rapidly evolving field.

To guide this review and adopt a shared understanding of micro-credentials, we subscribe to the following definition: *“A micro-credential is the record of the learning outcomes that a learner has acquired following a small volume of learning. These learning outcomes have been assessed against transparent and clearly defined standards. Courses leading to micro-credentials are designed to provide the learner with specific knowledge, skills, and competences that respond to societal, personal, cultural, or labour market needs. Micro-credentials are owned by the learner, can be shared and are portable. They may be standalone or combined into larger credentials. They are underpinned by quality assurance following agreed standards in the relevant sector or area of activity* [19]*.”* However, despite their growing popularity, there are a plurality of definitions across the general education literature (e.g., K-12), and conflicting data on their utility and impact [14,20,21,22,23]. Within health professions education, limited evidence exists on how micro-credentials are developed, implemented, and evaluated, and there remains little consensus on their scope, structure, and pedagogical foundations [24]. These uncertainties limit our ability to determine their value, viability, and potential impact on professional development. At worst, poorly designed micro-credentials may waste professionals’ time and financial resources, as well as institutional and faculty capacity, while falling short of their intended purpose: enhancing healthcare professionals’ knowledge and skills to ultimately improve patient care. Notably, despite this limited evidence base, significant investments have been made in micro-credentialing initiatives both in Canada and internationally [25,26,27,28,29], often without sufficient consideration of their pedagogical alignment, quality assurance, or evidence-informed educational principles. Therefore, the objective of this research is to map the breadth and depth of the existing literature on the use of micro-credentials in health professions education to clarify the current state of knowledge globally and inform application within a Canadian context.

## Methods

### Research co-production approach and knowledge user involvement

This project is grounded in a research co-production framework, where knowledge users actively participate as partners in the research process [30]. Since the results will inform an educational initiative for the respiratory therapy profession in Canada, representatives of the professional association (the Canadian Society of Respiratory Therapists) and its board of directors are included as key knowledge users [31]. Specifically, they collaborated in refining and aligning the objectives and research questions, shaped the methodology, and participated in the search, selection, data extraction, analysis and dissemination of the final results [30,32].

We used the six-stage methodological framework developed by Arksey and O’Malley [33], further refined by Levac and colleagues [34] and the JBI [35]. In keeping with the purpose of scoping reviews [36], we chose this type of review as it is designed to a) identify the types of available evidence in a given field, b) clarify key concepts/definitions in the literature, c) examine how research is conducted on a certain topic or field, and d) identify key characteristics or factors related to a concept. The full protocol was registered a priori in the Open Science Framework [37]. This study is reported following the Preferred Reporting Items for Systematic Reviews and Meta-Analyses-Extension for Scoping Reviews (PRISMA-ScR) checklist [38].

### Stage 1- Formulating the research question

The overarching research question guiding this review is: *what is known about the use of micro-credentials in health professions education?* The sub-questions include: 1) What are the instructional design features of micro-credentials in health professions education (e.g., cohort size, length, format)? 2) What pedagogical theories and approaches underpin the design of micro-credential programs in health professions education?; 3) What types of assessment strategies are used to evaluate learning outcomes?; 4) What are the reported impacts of using or implementing micro-credentials?

### Stage 2- Identifying the relevant literature

The research team worked with an academic research librarian (PF) to develop a database search strategy to retrieve records on micro-credentials in the context of health professions education. The search strategy was developed in MEDLINE and inspired by a previous review on the topic [39]. It was externally peer-reviewed by a second librarian according to the PRESS guidelines and then translated for other databases [40]. Supplemental File 1 includes the full search strategies. We systematically searched MEDLINE (Ovid), Embase (Ovid), CINAHL (EBSCOhost), ERIC (Ovid), Education Source (EBSCOhost), Scopus, and APA PsycINFO (Ovid) from the inception dates of the databases to December 2024. The references of the final included papers were hand-searched for additional relevant articles.

To complement the database searches, we conducted a structured grey literature search of Canadian sources. We reviewed the websites of institutions of higher education across all provinces in Canada that offered micro-credentials for healthcare professionals. Specifically, we conducted searches across the websites of U15 universities in Canada using each institution’s internal site search engine. The U15 is a group of Canada’s leading research-intensive universities committed to advancing research, innovation, and higher education. Commonly used search terms included “micro-credential,” “micro-credential AND health,” and “micro-credential AND continuing education.” When searches returned more than 50 results, only the first 50 were reviewed. This process was then repeated across all other Canadian universities with medical degree-granting faculties. Additionally, we searched the eCampus Ontario platform using its internal search filters to identify micro-credentials, further narrowing results with the keyword “health.” We then manually searched the websites of institutions identified as offering health-related micro-credentials. eCampus Ontario is a provincially funded organization that supports innovation, collaboration, and digital learning in Ontario’s post-secondary education system. Sources were included if their publicly available data addressed at least one of our research questions and specified a method of assessment. Grey literature searches were conducted between December 2024 and January 2025 and updated in May 2025.

We used the Population, Concept, and Context (PCC) framework to assess whether a paper was relevant to our research question and to define our inclusion and exclusion criteria [41]. The *population* for this review includes healthcare professionals. We define healthcare professionals as individuals formally recognized by a credentialling body as professionals who have passed and maintained all the qualifications to practice in that profession in a given state, province, or country. The list includes, but is not limited to, physicians, nurses, pharmacists, dietitians, social workers, clinical psychologists, and rehabilitation professionals (occupational therapists, physiotherapists, respiratory therapists, speech-language pathologists). We considered papers written in any language for inclusion and used a neural machine translation engine (DeepL) to translate articles into English to determine their eligibility.

The *concept* of interest is the use of micro-credentials to support continuous learning (i.e., CPD). We broadly define micro-credentials as short, competency-based certifications that validate and assess specific skills and knowledge aligned with industry or community needs. They are awarded by various providers, portable, and may complement larger qualifications. Micro-credentials are often used for upskilling and enhancing employability and are underpinned by quality assurance. We excluded records that described digital badges solely as visual indicators of participation or completion without formal assessment or credentialed learning, or records that described micro-learning strategies solely, defined as short, focused instructional content without associated certification or validation of learning outcomes.

The *context* for this review is continuing education in healthcare professions worldwide. Given that this review supports the development of an educational initiative for the respiratory therapy profession in Canada, we limited inclusion to papers that addressed at least one of the guiding research questions: (1) the design features of micro-credentials, (2) the pedagogical theories or approaches underpinning their development, (3) the assessment strategies used to evaluate learning outcomes, and (4) the reported impacts of implementing micro-credentials. Records that did not contribute substantively to at least one of these questions were excluded to ensure the relevance and applicability of findings to the intended knowledge user context. Supplemental File 2 includes the inclusion and exclusion criteria.

### Stage 3- Selecting the literature

Following the search, all identified records were imported into Covidence for duplicate removal and screening. The screening process consisted of two phases: a title and abstract review followed by a full-text review, carried out by three members of the research team (MZ, BD, ST).

For the title and abstract screening phase, all records were dual-screened. MZ served as the lead reviewer and screened all records, while BD and ST each independently screened 50% of the identified records. To ensure consistency, each reviewer pair (MZ+BD and MZ+ST) first completed a calibration exercise on 5% of the records. Reviewers independently screened these records and then met to discuss discrepancies. This process was repeated until a 90% agreement rate was reached [42]. Once the 90% agreement was reached, the remaining records were screened independently by each pair.

After screening all titles and abstracts, the team conducted full-text dual screening of the included papers using the same approach as previously described. In cases of disagreement, the reviewer pair discussed their decisions and attempted to reach consensus. If consensus could not be reached, the third team member resolved the conflict by reviewing the article and making the final decision.

### Stage 4- Data charting

The full research team participated in developing a data extraction form and mounted it onto Microsoft Excel. The charting form was based on existing micro-credential concept forms used in academic institutions [43,44,45]. The forms included information about each paper, including author(s), year of publication, country of origin, type of study, specific healthcare professional, design features of micro-credentials (e.g., delivery methods), gaps that the course aims to fill, learning outcomes, competencies, pedagogical theories/approach, assessment tools and information pertaining to the outcomes or impacts of micro-credentials (Supplemental File 3).

Although the original protocol outlined proposed dual independent data extraction following calibration, a pragmatic decision was made for the corresponding author (MZ) to complete data extraction for all included studies. This decision was based on resource limitations and the availability of team members during the review timeline. This approach is considered acceptable when justified, as noted in methodological references [41,46]. To maintain consistency, several safeguards were implemented: the extraction tool was structured and piloted to ensure clarity and reduce subjectivity, we documented all decisions systematically, and conducted regular check-ins with the two other reviewers (BD, ST) to review a subset of entries and clarify uncertainties. This approach promoted transparency and analytic rigour while balancing feasibility.

### Stage 5 – Reporting the results

Data analysis consisted of two phases: a numerical (i.e., bibliometric) analysis and qualitative content analysis [41]. The numerical analysis involved reporting the frequency and distribution of characteristics such as year of publication, country of origin, the targeted professions, terminology used to describe the micro-credentials, duration and cost to complete the micro-credential, and the modes of content delivery. The process of qualitative content analysis began with repeatedly reading all the excerpts to immerse oneself in the data. This is followed by reading excerpts word by word to capture key thoughts and concepts, and then using those concepts to generate the codes. We then organized the codes into categories based on how they were related and linked. Finally, we organized the categories into meaningful clusters.

Specifically, we also organized the extracted data related to the assessment strategies according to Miller’s Pyramid, which served as a guiding framework for classifying competency levels. While originally developed for assessing medical students and trainees, Miller’s Pyramid remains widely used across health professions, including in CPD [47]. This approach allowed us to identify whether micro-credential assessments primarily targeted knowledge acquisition or applied competencies.

### Stage 6- Consultation with knowledge users

This project is grounded in a co-production framework [30,32]. Two knowledge users (ST and AJW), who are members of the broader project steering committee, assumed more active leadership roles in this particular study. They contributed to the development of the research questions, data extraction and analysis, and interpretation of the findings. Additionally, BD, a practicing clinician, brought the end-user perspective to the team. Once the manuscript was drafted, we shared it, along with an executive summary of the results with the project steering committee. This group includes students and practicing professionals from various provinces and practice areas (e.g., education, clinical management, and bedside care). They were invited to review the findings and provide written feedback to ensure relevance and contextual accuracy. Their perspectives have been incorporated into the final manuscript.

The research team included both respiratory therapists and non-respiratory therapy members. While the project was conducted in the context of respiratory therapy to inform ongoing professional development initiatives, we deliberately structured the research questions and analytic process to ensure the findings would be applicable and relevant across health professions and educational contexts.

## Results

**Figure 1:**
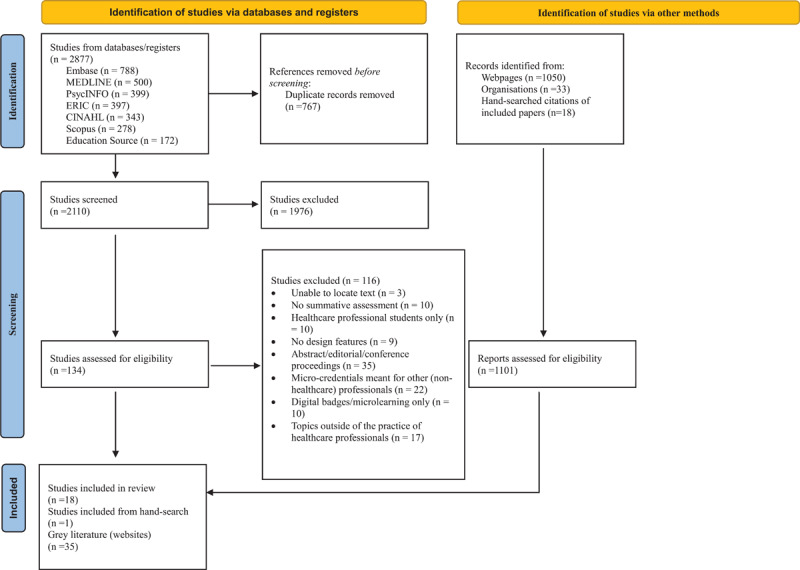
PRISMA flow diagram.

The results of the search are illustrated in the PRISMA flow diagram (Figure 1). The database search yielded 2877 papers. After removing 767 duplicates, we screened the titles and abstracts of 2110 papers, yielding 134 for full-text review. Of these, 18 met the inclusion criteria. One additional article was identified through hand-searching the reference lists of included studies, bringing the total number of included papers to 19.

For the grey literature, we conducted structured searches across the websites of U15 universities in Canada and across all other Canadian universities with medical degree-granting faculties, yielding 595 potential hits, from which 14 eligible institutional websites were included. Additional searches through eCampus Ontario produced another 455 potential hits, leading to the inclusion of 21. Full details are provided in Supplemental File 4.

Of note, all grey literature data were derived from publicly accessible institutional websites. In many cases, additional course details were restricted behind paywalls or paid registration portals, which prevented full access to course materials. These sources primarily consisted of promotional or descriptive summaries of micro-credential offerings, rather than formal evaluations or comprehensive program documentation. Consequently, data extraction was limited to surface-level features, such as delivery format, cost, and duration. Information about pedagogical theory/approach, assessment strategies, or learner outcomes was missing. In several cases, we were unable to confirm whether a formal assessment was included, and these micro-credentials were excluded.

### Numerical analysis

Table 1 summarizes the characteristics of the included papers. Most were published in 2024 (n = 8; 42.1%) and originated from the United States (n = 8; 42.1%). Nearly half of the studies (n = 9; 47.4%) did not employ a formal methodology and primarily consisted of descriptive accounts of micro-credential programs. Most micro-credentials were targeted at nurses (n = 11; 57.9%), followed by physicians (n = 6; 31.6%). No information about these characteristics was available for the grey literature.

**Table 1:** Demographics (n = 19)*.


	n (%)

**Year**	

2024	8 (42.1)

2023	3 (15.8)

2022	4 (21.1)

<2021	4 (21.1)

**Location**	

USA	8 (42.1)

Canada	5 (26.3)

Oceania	2 (10.5)

Europe	2 (10.5)

China	1 (5.3)

Middle East	1 (5.3)

**Research Approach**	

Quantitative	6 (31.6)

Qualitative	2 (10.5)

Mixed/Multimethod	2 (10.5)

Not described (e.g., description)	9 (47.4)

**Professions** ^+^	

Nursing	11 (57.9)

Medicine	6 (31.6)

Pharmacy	4 (21.1)

Social work	4 (21.1)

Public Health	3 (15.8)

Psychology	2 (10.5)

Allied health (non-specified)	2 (10.5)

Dentistry	1 (5.3)

Clinician-scientists	1 (5.3)

Midwifery	1 (5.3)

Nutrition	1 (5.3)

Occupational therapy	1 (5.3)

Physical therapy	1 (5.3)


**Note:** USA = United States of America. * Data includes peer reviewed literature only (all English publications). No data was available for grey literature. ^+^Studies could include more than one profession.

Table 2 summarizes the characteristics of micro-credentials described in the included papers and grey literature, where available. Most papers used the term micro-credential verbatim (n = 11; 57.9%), while some referred to digital badges (n = 4; 21.1%). Many micro-credentials focused on job- or discipline-specific topics (n = 14; 73.7%), including addiction medicine, bladder cancer management, gerontological care, global health, Indigenous cultural safety, and nighttime support for individuals living with dementia.

**Table 2:** Characteristics of Micro-credentials.


	PEER-REVIEWED PAPERS (n = 19)	GREY LITERATURE* (n = 35)

**Terminology**	**n (%)**	

Micro-credentials	11 (57.9)	29 (82.9)

Digital badges	4 (21.1)	0 (0)

Module certificate	2 (10.5)	0 (0)

Micro (learning) certificate	1 (5.3)	1 (2.9)

Mini certificate	1 (5.3)	0 (0)

Nano program	0 (0)	4 (11.4)

Self-paced certificate	0 (0)	1 (2.9)

**Identified skills**		

Other job/discipline-specific information	14 (73.7)	Not reported

Transferable skills (e.g., communication, problem solving, time management)	3 (15.8)	Not reported

Not reported	2 (10.0)	Not reported

	**Median (IQR)**	

**Length of time in hours**	n = 1516 (10.6 – 28.5)	n = 3235 (10.5 – 62.25)

**Cost (CAD)**	n = 7 $0 (0 – 91.51)	n = 28 $514 (218.75 – 905.75)

**Needs identified by partnerships**	**n (%)**	

Health institution employers	5 (26.3)	Not reported

University	3 (15.8)	Not reported

Implied but not defined	3 (15.8)	Not reported

Government	3 (15.8)	Not reported

Industry	2 (10.5)	Not reported

Knowledge users	2 (10.5)	Not reported

Professional Associations	1 (5.3)	Not reported

**Is micro-credential co-designed?**		

Yes	11 (57.9)	Not reported

No/Not reported	8 (42.1)	Not reported

**Details about instructors**		

Yes	8 (42.1)	Not reported

No/Not reported	11 (57.9)	Not reported

**Modes of delivery**		

Online only	8 (42.1)	0 (0)

Online and synchronous	0 (0)	4 (11.8)

Online and asynchronous/self-paced	6 (31.6)	22 (64.7)

Not reported	3 (15.8)	3 (8.8)

Online and social media	1 (5.3)	0 (0)

Hybrid (online and in-person)	1 (5.3)	6 (17.6)


**Note:** *Grey literature sources were institutional websites with limited publicly accessible information. Data restricted behind registration portals could not be accessed.

The training needs included in micro-credentials were most commonly identified by healthcare institutions/employers (n = 5; 26.3%). In over half of the papers, the micro-credentials were co-designed with the stakeholders who initially identified the need (n = 11; 57.9%). Most programs were delivered exclusively online (n = 8; 42.1%).

Information about instructors or facilitators was often lacking, with over half of the papers not specifying who led the training (n = 11; 57.9%). Among the peer-reviewed papers, micro-credentials were typically offered free of charge to participants under study (n = 7; median cost = $0 [IQR $0–96.75]) and required a median of 16 hours to complete (n = 15; IQR 10.6–28.5 hours). In contrast, the grey literature, which consisted of institutional websites, more commonly described micro-credentials that were delivered online and asynchronously (n = 22; 68.8%), with a median cost of $514 (n = 28; IQR $218.75–905.75) and a median duration of 35 hours (IQR 10.5–62.25 hours).

### Qualitative content analysis

We conducted a qualitative content analysis of the included peer-reviewed and grey literature sources to address our four research questions. Using inductive data analysis, we systematically extracted and coded descriptive text related to the following categories: (1) design features, (2) pedagogical theories/approaches underpinning micro-credentials, (3) assessment strategies, and (4) reported impacts. Codes were grouped into categories based on recurring patterns and features across studies.

#### Instructional design features of micro-credentials: Variety is the spice of life

The instructional design features of micro-credentials refer to the specific strategies and formats used to deliver content and facilitate learning. In the peer-reviewed papers, 14 (73.7%) reported at least one design feature. We identified a wide range of instructional design features, reflecting a high degree of variability across the micro-credential reporting articles and a lack of a standardized rationale guiding the choice of instructional modalities. Instead, developers appeared to draw from a diverse range of design options, akin to a “buffet” of educational approaches.

Common instructional design elements include multimedia materials, resource documents to read, embedded self-reflection activities, and varied module structures. For example, some micro-credentials (n = 3; 15.8%) allowed learners to complete modules in any order [48,49,50], while others (n = 4; 21.1%) followed a prescribed sequence [51,52,53,54]. Similarly, while some (n = 3; 15.8%) incorporated synchronous discussions [51,52,54], others (n = 5; 26.3%) relied exclusively on asynchronous delivery [53,55,56,57]. The most commonly reported design feature was team-based active learning (n = 8; 42.1%). Examples included collaborative games with group debriefs, assigning learners to teams to complete portions of the micro-credential together (e.g., preparing and delivering a joint presentation), and organizing virtual colloquia to facilitate meaningful interaction between learners and subject matter experts. The second most frequent design element was the use of multimedia learning tools, such as simulation videos, audio-visual materials, animated content, educational games, and podcasts (n = 7; 36.8%). Finally, many micro-credentials were supplemented with written resources, including course readings, scholarly articles, and curated websites, to reinforce and extend learning. Overall, these design choices appeared to be guided more by practical constraints and developer intuition rather than grounded in a consistent pedagogical or theoretical rationale. While such diversity may reflect responsiveness to learner needs, it also raises questions about the coherence and reproducibility of design decisions across programs.

In the grey literature (n = 35 online course descriptions), design features were generally less detailed. Most websites provided only broad descriptions of format. The majority of micro-credentials were described as online and asynchronous (n = 22; 64.7%), followed by hybrid delivery combining online modules with occasional in-person training (e.g., wet labs, simulation labs) (n = 4; 11.8%), and online synchronous formats (n = 4; 11.8%). Three listings (8.8%) did not specify a delivery mode. Overall, the lack of detailed design information in the grey literature limited our ability to meaningfully compare features across institutional offerings or peer-reviewed literature, which is why they are reported separately. When considered alongside peer-reviewed studies, both sources reveal substantial variability in instructional approaches and a lack of standardized pedagogical rationale, highlighting the descriptive rather than evaluative nature of current reporting.

#### Micro-credentials are pedagogically undertheorized

We examined the extent to which the included studies described the pedagogical theories/approaches which inform the design of micro-credentials. Across the articles, these pedagogical theories/approaches were not explicitly stated. However, a small number of papers (n = 4; 21.1%) made direct reference to underpinning educational philosophies. These included constructivism, which views learning as an active process of building knowledge; social constructivism, which emphasizes the role of social interaction and collaboration in shaping learning; cognitive load theory, which highlights the importance of managing learners’ mental processing capacity; developmentalism, which acknowledges that learners’ readiness and cognitive abilities evolve over time and that instruction should be adapted accordingly; and meaningful learning, which suggests that knowledge is best acquired when it connects to learners’ prior understanding and is relevant to real-world contexts.

In several papers (n = 6; 31.6%), pedagogical principles were implied through descriptions of instructional strategies, such as active learning, critical pedagogy, Indigenous pedagogy or team-based activities, but were not explicitly attributed to any specific pedagogical theory. Overall, explicit discussion of pedagogical theory and/or approach was limited, and when present, was often brief and lacked elaboration on how the philosophy informed instructional choices, learner engagement, the design or delivery of the micro-credential, or assessment strategies. No information about pedagogical principles was described in the grey literature.

#### Assessment in micro-credentials: Focused on knowing

**Figure 2:**
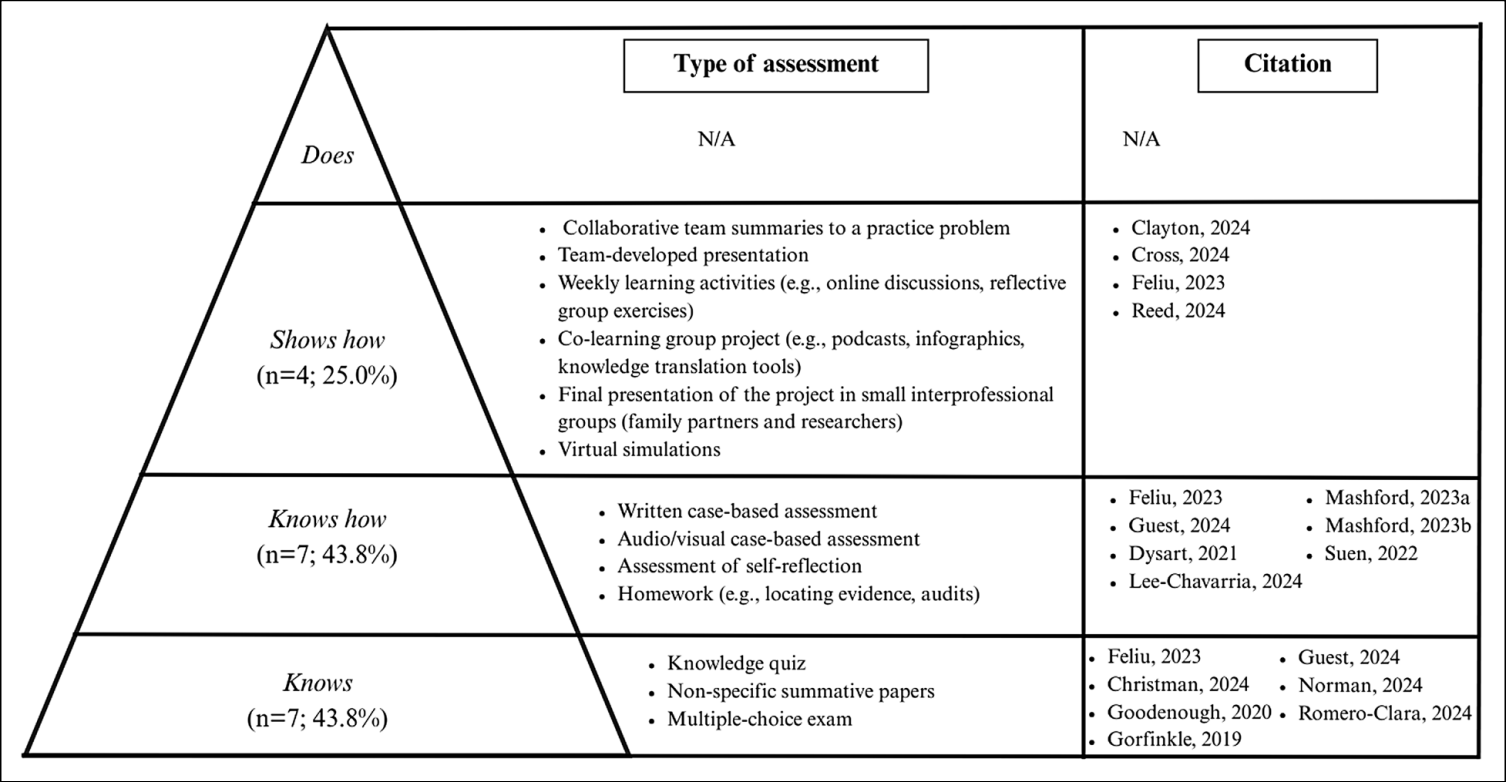
Micro-credential assessments according to Miller’s Pyramid of Clinical Competence. **Note:** Peer-reviewed papers could have multiple assessments, so they are in multiple levels (n = 16). Not enough information provided in grey literature to plot in Figure.

Assessment strategies across the reviewed peer-reviewed papers primarily emphasized summative assessment (i.e., assessment *of* learning) methods, with a focus on knowledge-check tasks such as multiple-choice questions and quizzes [48,49,50,55,58,59,60,61]. We organized these summative assessments according to Miller’s Pyramid of Clinical Competence as a guiding framework [47] (Figure 2). In the context of assessment, Miller’s Pyramid provides a structured framework for evaluating competence, progressing from assessing factual knowledge (*knows*), to applied understanding (*knows how*), to demonstration of skills in controlled settings (*shows how*), and ultimately to performance in real-world practice (*does*). The summative assessments described in the papers were predominantly situated at the “*knows*” and “*knows how*” levels. In fewer cases (n = 4; 25.0%), assessments moved beyond knowledge recall to evaluate higher-level competencies, such as “*shows how*” through simulation or team-based tasks. For example, defining a problem as a team, generating an executive summary, and presenting it as a team [62]. Another example includes teams co-creating a knowledge translation tool or resource on family engagement in research (e.g., podcasts, videos, infographics) and presenting the resource [54]. No summative assessments were categorized under the “*does*” level.

Formative assessment (i.e., assessment *for* learning) strategies, such as self-reflection, discussion forums, and participating in synchronous and asynchronous peer discussions were occasionally used to support learner progression. This approach to assessment *for* learning provides deeper insight into what learners know and how they use it. This enables more effective modification of learning activities to engage learners and address individual student learning needs [63]. Additionally, few (n = 2; 10.5%) papers described the use of collaborative assessment, defined as an approach that incorporates reciprocal feedback between instructors and students or among peers. In these cases, assessment involved working in teams, co-creating knowledge translation tools, or engaging in peer “teach-back” activities [52,54]. One paper (5.3%) made mention equity-oriented assessment methods, which allowed learners some control over how they were assessed, for example, by selecting their own project as part of the assessment to earn the micro-credential [62]. Overall, assessment practices in micro-credentials were largely weighted toward knowledge acquisition.

Assessment practices in the grey literature mirrored those in peer-reviewed sources, focusing primarily on knowledge acquisition. The most frequently reported strategy was the use of knowledge quizzes, which appeared across the majority of institutional listings (n = 24; 68.6%). Other approaches included self-assessment activities (n = 5; 14.3%), lab or simulation components (n = 5; 14.3%), written case projects (n = 3; 8.6%), and assessments tied to participation in discussion forums or reflective exercises (n = 2; 5.7%). In most cases, assessment strategies were listed in the course description but lacked detailed explanations of their purpose or alignment with learning outcomes. Across sources, assessment within micro-credentials remains largely limited to lower levels of competence and provides minimal insight into learners’ ability to apply or sustain skills in practice.

#### Impact of micro-credentials: unclear value of the format

Among the included papers, 11 (57.9%) did not report any measurable or descriptive impacts. The remaining eight papers (42.1%) described some form of impact, though the nature of these impacts varied. Most commonly, reported outcomes pertained to improvements in knowledge acquisition, learner confidence, or engagement with the subject matter. For example, some studies documented increased post-test scores, positive learner feedback, or perceived relevance of the content to professional practice. However, these impacts were specifically tied to the educational content delivered rather than to the micro-credential format itself. A small subset of authors offered reflections that indirectly referenced the delivery model, such as micro-credentials’ flexible learning, or its modular design, [48,50] which could be beneficial. However, these comments were anecdotal and not the focus of a formal evaluation. There was no evidence that directly assessed or attributed impact to the micro-credential as a distinct educational format. Similarly, grey literature sources provided no evidence of outcome measurement beyond general promotional statements.

## Discussion

The objective of this review was to map the breadth and depth of existing literature on the use of micro-credentials in health professions education. While many of the included papers described the content and structure of micro-credentials, few evaluated which design features were most effective. Some studies reported participants’ outcomes such as increased knowledge, confidence, or engagement with a topic; however, these outcomes were typically attributed to the educational content of the topic rather than to the micro-credential format itself. While micro-credentials are often promoted as a response to the limitations of traditional CPD [7,8,9,10,11], the findings of this review suggest that many of those challenges remain insufficiently addressed. Micro-credentials were frequently designed to improve flexibility, accessibility, and offer personalized learning, yet few papers described how these design features translated into meaningful or sustained learning. Although their modular and online formats offer practical advantages for busy clinicians, most papers lacked detail on mechanisms that foster ongoing competency development, reflection, or transfer of learning into practice. Furthermore, the predominance of short, knowledge-focused assessments suggests that micro-credentials may replicate rather than resolve the issues of superficial engagement and limited impact on professional performance identified in earlier CPD literature [6,7,9].

Overall, the findings of this review reflect what is currently being reported in the literature, rather than evidence of what works best. This is particularly concerning given existing reviews on teaching modalities, which consistently find no single instructional approach to be clearly superior in improving learner competence [64,65,66,67]. These findings raise important questions about the added value of micro-credentials as a delivery format. Future research should focus on conducting evaluative studies to determine whether micro-credentials (as a distinct modality) improve professional competencies in meaningful ways.

There was frequently a misalignment between the instructional strategies and assessment practices in both the peer-reviewed papers and the grey literature. While many micro-credentials incorporated active and applied learning approaches, including simulation videos, animated content, educational games, podcasts, group work, and reflective exercises, they relied primarily on traditional assessment of learning strategies, such as multiple-choice quizzes, or knowledge checks to assess learning. When analyzed according to Miller’s Pyramid of Clinical Competence [47], most assessments targeted the lower levels (“*knows*” and “*knows how*”), with far fewer addressing the higher levels (“*shows how*” or “*does*”), which are critical for evaluating real-world professional competence. This disconnect limits the potential of micro-credentials to support meaningful competency development for practicing professionals.

Some authors have argued that assessments focused on lower-level knowledge recall (e.g., assessment *of* learning strategies) no longer align with contemporary definitions of professional competence in the health professions, which emphasize applied skills, critical thinking, and real-world performance [68,69]. A small number of papers suggested alternative assessment approaches, such as sustainable assessment and equity-oriented assessment, that may better align with the goals of micro-credentials. These approaches reflect key values often associated with micro-credentials, including personalization, accessibility, and a focus on practical, competency-based learning to upskill workers to reflect current workforce needs [19,45,70,71,72]. Moreover, they align with the idea of assessment *as* learning, a subset of assessment *for* learning, that emphasizes the use of assessment for developing metacognition in learners [63]. For example, sustainable assessment refers to assessment that meets students’ immediate learning needs and prepares them to manage their own future learning [73,74]. This includes practices like peer feedback and self-reflection, which help learners develop lifelong learning skills, self-regulation, and the capacity for reflective practice, important competencies for healthcare professionals. Similarly, equity-oriented assessment prioritizes creating multiple ways for learners to demonstrate what they know and can do. This approach acknowledges diverse learner needs, backgrounds, and contexts and may involve offering alternative modes of assessment (e.g., oral versus written formats, asynchronous versus synchronous delivery) or enabling learners to complete community- or workplace-based projects. Such approaches are increasingly being discussed within health professions education and may offer more inclusive and authentic ways to assess competence in micro-credentials [75,76].

Overall, the lack of clarity about whether micro-credentials represent genuine pedagogical innovation warrants attention. Micro-credentials are presented as modern, accessible, promoting inclusive and equal opportunities and transformative for higher education [19,77,78]. However, it remains unclear whether they offer a fundamentally new approach to education or simply repackage traditional CPD under a new label, a concern raised by others [26,79,80]. This aligns with findings from past reviews, which caution against overstating the impact of specific educational formats [64,65,66,67]. Despite these concerns, micro-credential offerings continue to grow rapidly in health professions education and other fields (e.g., technology, engineering, higher learning). However, the evidence base supporting their educational value remains limited, underscoring the need for rigorous studies to determine their effectiveness relative to traditional CPD approaches. Articles published in *University Affairs* [25,26], a widely recognized source on Canadian postsecondary education, and empirical studies by authors such as Peters [79] and Ralston [80] suggest that this expansion might be driven more by institutional trends and market demand than by evidence of educational benefit or value [27,28,29,81,82]. This remains an important area for future research.

Despite the lack of explicitly stated educational theories in the design of micro-credentials across the included papers, our findings allow for some important inferences that can help advance the body of knowledge. In the few papers that referenced theoretical foundations (i.e., constructivism, social constructivism, meaningful learning, developmentalism, and cognitive load theory) and in the instructional strategies described across studies, we observed an implicit alignment with established pedagogical principles. Based on these observations, we suggest that the pedagogical underpinnings of micro-credentials in health professions education can be understood as a composite framework informed by multiple formal learning theories. This inferred foundation conceptualizes learning as a constructive, context-sensitive, and stage-dependent process. Specifically, learners build knowledge by engaging with their clinical experiences (reflecting constructivism). The learning should be connected to the learner’s clinical, cultural, and professional context (aligning with the principles of social constructivism and situated learning). Finally, recognizing that learners have different levels of experience, learning should be a stage-based approach that uses scaffolding to tailor instruction to their professional stage, making it more relevant.

### Strengths and limitations

This review has several strengths but is not without limitations. The search strategy was robust, developed in collaboration with an academic research librarian and externally peer-reviewed to ensure comprehensiveness and methodological rigor. The peer-reviewed literature captured international perspectives, ensuring that findings are not solely reflective of the Canadian context. For the grey literature, we included only publicly available webpages that described assessment criteria. While this may not capture all existing micro-credential offerings, it provides a strong snapshot of current practice.

The grey literature was limited to Canadian sources due to the co-production foundation of the study, which may affect the transferability of findings to other countries with different educational systems. However, the co-designed nature of the study is a strength, ensuring the findings are relevant, actionable, and useful for guiding the future development and evaluation of micro-credentials in health professions education.

Consistent with scoping review methodology, we did not conduct a formal quality appraisal of included studies, as our focus was on mapping the breadth and nature of the evidence rather than evaluating its quality. This limits our ability to distinguish methodologically rigorous studies from descriptive accounts, though this limitation reflects the current state of the field, where nearly half (47.4%) of identified papers lacked a formal research methodology.

Finally, this review focused specifically on practicing healthcare professionals, where micro-credentials remain an emerging area of research. As such, the findings may not apply to other disciplines, such as engineering or law, or be directly applicable to healthcare professional students. Finally, although the proposed composite framework does not constitute a formal theory, it integrates several well-established educational perspectives and provides a useful lens for understanding current micro-credentials and guiding their future design. Similarly, the assessment component of our analysis was guided by Miller’s Pyramid, which remains a widely used framework for categorizing competency levels in health professions education. Nonetheless, frameworks specifically designed for CPD may provide additional insights for evaluating micro-credentials in professional learning contexts.

## Conclusion

Micro-credentials are gaining traction as a unique approach to CPD for practicing healthcare professionals, intended to offer a personalized, flexible approach to learning by enabling clinicians to acquire targeted skills aligned with their specific roles and goals. However, this review highlights limited evidence on how they are developed, implemented, and evaluated in health professions education. Most do not make their pedagogical foundations evident, though they implement a wide range of instructional methods in their designs. The approaches to assessment in micro-credentials often focus on evaluating lower-level knowledge rather than on those that support applied competency or metacognitive development. We propose that their underlying pedagogical foundation can be understood as a composite framework of established learning theories, framing learning as a constructive, context-sensitive, and stage-based process. We found no strong evidence that micro-credentials, as a teaching modality, offer distinct educational benefits.

## Additional File

The additional file for this article can be found as follows:

10.5334/pme.2038.s1 Supplemental Files. Supplemental Files 1, 2, 3 and 4.

## Artificial Intelligence Use

ChatGPT 4o was used to refine grammar. No content was generated by artificial intelligence.

## REB Requirement

Given that this work relied on publicly available document, no REB approval was necessary.
