# Supplementary material for: The Use of Micro-Credentials in Health Professions Education: A Scoping Review
Source: Perspect Med Educ. 2025 Dec 5;14(1):974–90. doi: 10.5334/pme.2038 (PMC12680006; doi:10.5334/pme.2038)
Supplement: Supplemental Files. — Supplemental Files 1, 2, 3 and 4. [file pme-14-1-2038-s1.pdf]

Supplemental File 1: Database search strategies

Summary

Database	Platform	Update	Date searched	Notes
MEDLINE	Ovid	Ovid MEDLINE(R) ALL <1946 to December 18, 2024>	2024-12-19	
Embase	Ovid	Embase Classic+Embase <1947 to 2024 December 18>	2024-12-19	
APA PsycInfo	Ovid	APA PsycInfo <1806 to December 2024 Week 2>	2024-12-19	
CINAHL	Ebsco	1981 to December 18, 2024	2024-12-19	
ERIC	Ovid	1965 to November 2024	2024-12-19	Health concept was added to limit education database to medical education
Education Source	Ebsco	na	2024-12-19	Health concept was added to limit education database to medical education
Scopus	Scopus	na	2024-12-19	Health concept was added to limit multidisciplinary database to health subjects

Ovid MEDLINE(R) ALL

1	(minibadg* or microcredential* or "micro credential*" or nanocredential or "nano credential*" or "nano degree" or nanodegree or "minicredential*" or "mini credential*" or "digital credential*" or "digital badg*" or microcertificat* or "micro certificat*" or nanocertificat* or "nano-certificat*" or minicertificat* or "mini certificat*" or digital certificate? or microlearn* or micro learn*).ti,ab,kf.
2	(badge or badges or badging or "online certificat*").ti,ab,kf.
3	education.ti,ab,kf,fs.
4	exp credentialing/
5	(professional development or learn* or train* or CPD or CPE or

	CME).ti,ab,kf.
6	2 and (3 or 4 or 5)
7	1 or 6

Embase Classic+Embase

1	(minibadg* or microcredential* or "micro credential*" or nanocredential or "nano credential*" or "nano degree" or nanodegree or "minicredential*" or "mini credential*" or "digital credential*" or "digital badg*" or microcertificat* or "micro certificat*" or nanocertificat* or "nano-certificat*" or minicertificat* or "mini certificat*" or digital certificate? or microlearn* or micro learn*).ti,ab,kf.
2	(badge or badges or badging or "online certificat*").ti,ab,kf.
3	education.ti,ab,kf.
4	exp credentialing/
5	(professional development or learn* or train* or CPD or CPE or CME).ti,ab,kf.
6	2 and (3 or 4 or 5)
7	1 or 6

**APA PsycInfo**	
1	(minibadg* or microcredential* or "micro credential*" or nanocredential or "nano credential*" or "nano degree" or nanodegree or "minicredential*" or "mini credential*" or "digital credential*" or "digital badg*" or microcertificat* or "micro certificat*" or nanocertificat* or "nano-certificat*" or minicertificat* or "mini certificat*" or digital certificate? or microlearn* or micro learn*).ti,ab.
2	(badge or badges or badging or "online certificat*").ti,ab.
3	education.ti,ab.

4	exp Professional Development/
5	(professional development or learn* or train* or CPD or CPE or CME).ti,ab.
6	2 and (3 or 4 or 5)
7	1 or 6

CINAHL

((TI(badge OR badges OR badging OR "online certificat*") OR AB(badge OR badges OR badging OR "online certificat*")) AND ((TI(education) OR AB(education)) OR ((MH "Credentialing+")) OR (TI(professional development OR learn* OR train* OR CPD OR CPE OR CME) OR AB(professional development OR learn* OR train* OR CPD OR CPE OR CME)))) OR (TI(minibadg* OR microcredential* OR "micro credential*" OR nanocredential OR "nano credential*" OR "nano degree" OR nanodegree OR "minicredential*" OR "mini credential*" OR "digital credential*" OR "digital badg*" OR microcertificat* OR "micro certificat*" OR nanocertificat* OR "nano-certificat*" OR minicertificat* OR "mini certificat*" OR digital certificate? OR microlearn* OR micro learn*) OR AB(minibadg* OR microcredential* OR "micro credential*" OR nanocredential OR "nano credential*" OR "nano degree" OR nanodegree OR "minicredential*" OR "mini credential*" OR "digital credential*" OR "digital badg*" OR microcertificat* OR "micro certificat*" OR nanocertificat* OR "nano-certificat*" OR minicertificat* OR "mini certificat*" OR digital certificate? OR microlearn* OR micro learn*))

ERIC	
1	(minibadg* or microcredential* or "micro credential*" or nanocredential or "nano credential*" or "nano degree" or nanodegree or "minicredential*" or "mini credential*" or "digital credential*" or microcertificat* or "micro certificat*" or nanocertificat* or "nano-certificat*" or minicertificat* or "mini certificat*" or digital certificate? or microlearn* or micro learn*).ti,ab.
2	(badge or badges or badging or "online certificat*").ti,ab.
3	credentials/ or Educational Certificates/ or Microcredentials/

4	(health or healthcare or interprofessional or IPE or medical or medicine or physician* or surge* or nurse* or nursing or pharmacy or pharmacist* or dental or dentistry or dietitian* or social work* or clinic* or hospital or psychologist or therapist* or physiotherap* or patholog* or audiolog* or paramedic* or radiolog* or doctor* or vet* or perfusion* or speech*).ti,ab,hw.
5	2 and (3 or 4)
6	1 or 5

Education Source

s1	TI(minibadg* or microcredential* or "micro credential*" or nanocredential or "nano credential*" or "nano degree" or nanodegree or "minicredential*" or "mini credential*" or "digital credential*" or "digital badg*" or microcertificat* or "micro certificat*" or nanocertificat* or "nano-certificat*" or minicertificat* or "mini certificat*" or digital certificate? or microlearn* or micro learn*) OR AB(minibadg* or microcredential* or "micro credential*" or nanocredential or "nano credential*" or "nano degree" or nanodegree or "minicredential*" or "mini credential*" or "digital credential*" or "digital badg*" or microcertificat* or "micro certificat*" or nanocertificat* or "nano-certificat*" or minicertificat* or "mini certificat*" or digital certificate? or microlearn* or micro learn*)
s2	TI(badge or badges or badging or "online certificat*") OR AB(badge or badges or badging or "online certificat*")
s3	DE "Digital badges in education"

S4	TI(health or healthcare or interprofessional or IPE or medical or medicine or physician* or surge* or nurse* or nursing or pharmacy or pharmacist* or dental or dentistry or dietitian* or social work* or clinic* or hospital or psychologist or therapist* or physiotherap* or patholog* or audiolog* or paramedic* or radiolog*) OR AB(health or healthcare or interprofessional or IPE or medical or medicine or physician* or surge* or nurse* or nursing or pharmacy or pharmacist* or dental or dentistry or dietitian* or social work* or clinic* or hospital or psychologist or therapist* or physiotherap* or patholog* or audiolog* or paramedic* or radiolog*) OR SU(health or healthcare or interprofessional or IPE or medical or medicine or physician* or surge* or nurse* or nursing or pharmacy or pharmacist* or dental or dentistry or dietitian* or social work* or clinic* or hospital or psychologist or therapist* or physiotherap* or patholog* or audiolog* or paramedic* or radiolog*)
S5	(S1 OR S2 OR S3) AND S4

Scopus

(TITLE-ABS-KEY (health OR healthcare OR interprofessional OR ipe OR medical OR medicine OR physician* OR surge* OR nurse* OR nursing OR pharmacy OR pharmacist* OR dental OR dentistry OR dietitian* OR social AND work* OR clinic* OR hospital OR psychologist OR therapist* OR physiotherap* OR patholog* OR audiolog* OR paramedic* OR radiolog* OR doctor* OR vet* OR animal* OR perfusion* OR speech*)) AND ((TITLE-ABS-KEY (badge OR badges OR badging OR "online certificat*") AND TITLE-ABS-KEY (professional AND development OR learn* OR train* OR cpd OR cpe OR cme OR education)) OR TITLE-ABS-KEY (minibadg* OR microcredential* OR "micro credential*" OR nanocredential OR "nano credential*" OR "nano degree" OR nanodegree OR "minicredential*" OR "mini credential*" OR "digital credential*" OR "digital badg*" OR microcertificat* OR "micro certificat*" OR nanocertificat* OR "nano-certificat*" OR minicertificat* OR "mini certificat*" OR digital AND certificate? OR microlearn* OR "micro learn*"))

Supplemental File 2-Inclusion and exclusion criteria for empirical and grey literature

Inclusion	Exclusion
• Papers that describe the use of micro-credentials in the context of postgraduate health professions education or professional development.	• Papers about micro-credentials for non-healthcare professions learners<br>• Papers about micro-credentials for healthcare professions learners only
• Any methodology: quantitative, qualitative, knowledge syntheses and mixed methods.	• Non-empirical papers (e.g., conference abstract, commentaries and letters to the editors)<br>• Inability to access papers
• Any paper published as of 2005, which is the date when the internet became readily available. Since micro-credentials are designed to offer professionals flexible learning options, it is reasonable to include those that incorporate online components.	• Papers addressing topics within micro-credentials that are outside the practice of healthcare professionals<br>• Papers about micro-credentials meant for other (non-healthcare) professionals
• Any healthcare professionals possessing a credential to practice in that profession, in a given state, province, or country	• Described digital badges without assessment
• Published in any language	• Described micro-learning without assessment
• Addresses at least one of the research questions	• Papers with no design features<br>• Papers with no assessment of knowledge

Supplemental File 3: Draft data extraction form

Author	
Title of publication	
Year of publication	
Geography	
Type of article (e.g., conceptual, empirical, position paper)	
Study objective	
Study design	
Methodology (Qual, Quan, Mixed methods or N/A)	
Profession(s)	
What is degree called?	
Definitions provided	
Cost	
Sample Population (n=)	
Q1) Design features of micro-credential	
• Length of micro-credential	
• Co-designed with stakeholders	
• Details about instructors	
• Format (e.g. online, face-to-face, hybrid)	
Q2) What pedagogical approaches underpin the design?	
• Learning outcomes	
• Listed competencies	
Q3) What types of assessments are used?	
Q4) What are the impacts/outcomes of micro-credentials?	
What skill gaps do the micro-credential fill?	
Any partner/industry endorsement?	
Any discussion of barriers/facilitators?	
Authors results/main conclusion	

Study limitations	
Areas for future research	

Supplemental File 4- Grey literature search

**Searched all U15 schools for micro-credentials (https://u15.ca)**							
**Date**	**Organization Name**	**URL**	**Search strategy(s)/ words searched including (if applicable) how items were selected.**	**# items retrieved/ search results**	**Included?**	**Reasons for exclusion**	**Interesting papers/ comments**
Dec. 29, 2024	University of Alberta	https://www.ualberta.ca/en/search/index.html#q=microcredentials&t=Main&sort=relevancy	1) "micro-credential"	1) 104 (reviewed the first 50 hits)	Yes<br>1) College of rehab: https://www.ualberta.ca/en/rehabilitation/programs/professional-development/micro-courses/index.html		8 micro-credentials in rehab.
Dec. 29, 2024	University of Saskatchewan	https://search.usask.ca/index.php?q=%22micro-credentials%22+and+%22health%22	1) "micro-credential" and health"	1) 368 results on 10 pages (screened 50 on first 5 pages)	No (updated. May 23, broken link) https://nursing.usask.ca/programs/micro-credentials/micro-credentials.php		https://news.usask.ca/articles/colleges/2021/usask-moves-forward-with-microcredentials.php
Dec. 29, 2024	University of British Columbia	https://www.ubc.ca/search/?q=micrcredeNtIAl#gsc.tab=0&gsc.q=%22microcrede	1) "Micro-credential" and "health"	1) 14 (screened all)	Yes)<br>1) https://extendedlearning.ubc.ca/programs-credentials/health-data-		

		NtIAl%22%20and%20%22health%22&gsc.sort=			analytics-opportunities-applications-microcertificate		
Dec. 29, 2024	University of Calgary	https://www.ucalgary.ca/sitesearch?scope=global&m=&as_q=micro-credentials	1) "Micro-credential" and "health"	1) 55 (screened first 50)	Yes<br>1) https://nursing.ucalgary.ca/teaching-and-learning/pep/nursing-simulation-education		Part of $500 million to increase micro-credential: https://www.ucalgary.ca/sites/default/files/teams/157/2022-23%20Annual%20Report.pdf<br><br>Steps to create a micro-credential: https://nursing.ucalgary.ca/sites/default/files/teams/20/7_Steps_to_Creating_a_Micro-credential.pdf<br><br>Interesting article: https://ucalgary.ca/news/modular-education-offers-innovative-advantages-competitive-labour-market
Dec. 29, 2024	Dalhousie University	https://www.dal.ca/faculty/open/microcredentials.html	1) "micro-credential"	1) 9	No	Not focused on HPE	https://www.dal.ca/faculty/open/microcredentials.html
Dec. 29, 2024	Université de Laval	https://www.ulaval.ca/etudes/nanoprogrammes	1) "nanoprogramme"	1) 243 (reviewed the first 50 hits)	Yes<br>1) 4 all pharmacy: https://www.ulaval.ca/etudes/nanoprogrammes		

Dec. 29, 2024	University of Manitoba	N/A	1) "Micro-credential" and "health"	1) 30	No	Not focused on HPE	
Dec. 29, 2024	McGill University	https://www.mcgill.ca/search/?query=microcredential&search_origin=continuingstudies	1) microcredential	1) 48	No	Not focused on HPE	
Dec. 29, 2024	McMaster University	N/A	1) "Micro-credential" and "health"	1) 54 (screened first 50)	No<br>1) health research methodology https://continuing.mcmaster.ca/programs/health-social-services/fundamentals-of-health-research-methodology/	No assessment mentioned	https://continuing.mcmaster.ca/programs/microcredentials/
Dec. 29, 2024	Université de Montréal	https://admission.umontreal.ca/rechercher/?q=microcredential	1) "micro-credential"<br>2) "nanoprogramme"	1) 0<br>2) 0	No	N/A	
Dec. 30, 2024	University of Ottawa	https://nursing.ucalgary.ca/teaching-and-learning/pep/nursing-simulation-education	1) "microcredential"<br>2) "microprogram" and "health"<br>3) "microprogramme"	1) 3<br>2) 1440 (searched first 50)<br>3) 2150 (searched first 50)	No	No assessment method mentioned	https://www.uottawa.ca/study/graduate-studies/microprograms<br><br>https://catalogue.uottawa.ca/en/programs/#filter=.filter_183

Dec. 30, 2024	Queen's University	https://www.queensu.ca/microcredentials/	1) "micro-credential"	1) 7	No	No assessment method mentioned	https://www.queensu.ca/microcredentials/collection/12
Dec. 30, 2024	University of Toronto	https://www.utoronto.ca/search?query=microcredential#gsc.tab=0&gsc.q=microcredential&gsc.page=1	1) "micro-credential"	1) 5	No	Not focused on HPE	https://learn.utoronto.ca/programs-courses/unique/micro-courses-and-micro-credentials
Dec. 30, 2024	University of Waterloo		1) "Micro-credential" and "health"	1) 22	No	Not focused on HPE	https://uwaterloo.ca/work-learn-institute/value-microcredentials-next-gen-talent<br><br>https://uwaterloo.ca/renison-continuing-education/micro-credentials
Dec. 30, 2024	Western University	https://cse.google.com/cse?cx=013266746113315494840%3Axq6zhlrbloa&as_sitesearch=&q=micro-credential#gsc.tab=0&gsc.q=%22micro-credential%22%20and%20%22health%22&gsc.sort=	1) "Micro-credential" and "health"	1) 94 (first 50)	No	Not focused on HPE	https://wcs.uwo.ca/contentManagement.do?method=load&code=CM000388<br><br>https://wcs.uwo.ca/contentManagement.do?method=load&code=CM000034

**Then searched the rest of medical degree granting institutions (https://en.wikipedia.org/wiki/List_of_medical_schools_in_Canada)**							
Dec. 29, 2024	Memorial University of Newfound Land	https://www.mun.ca/search/?q=MICRO-CREDENTIAL	1) "Micro-credential" and "health"<br>2) "Micro-credential"	1) 6<br>2) 112 on 10 pages (screened first 3 pages n=28)	No	Not focused on HPE	https://www.mun.ca/gardinercentre/about-us/digital-badging-and-micro-credentials/<br>Ministers Investing $634k in 2024 to build micro-credentials to help upskill participants in tech sector: https://www.mun.ca/ciap/media/production/ciap/media-library/planning/MUNAnnualReport2023-24.PDF
Dec. 30, 2024	Northern Ontario School of Medicine	https://www.nosm.ca/?s=micro-credential	1) "micro-credential"	1) 1	No	Not focused on HPE	
**Then I went to eCampus Ontario and used their search function https://microlearnontario.ca/mc-search/<br>Used the filter to focus only on micro-credential and all keyword "health" to identify schools that did micro-credentials and then searched in those schools**							
Dec. 30, 2024	Laurentien University		1) "micro-credential"	1) 8	Yes	3 courses, sylliby have assessments	https://laurentian.ca/continuing-learning/course-offerings#micro-credential
Dec. 30, 2024	Mohawk College		1) "Micro-credential" and "health"	1) 6826 (screened first 50)	No	No assessment method mentioned	https://cereg.mohawkcollege.ca/search/publicCourseCertificateSearch.do;jsessionid=4A59097275DF51BB6396A951A2817B70?method=doFilter&t

							ype=subject&objectId=1009671
Dec. 30, 2024	Seneca Polytechnic	https://www.senecapolytechnic.ca/search.html?q=%22micro-credential%22	1) "micro-credential"	1) 10	No	No assessment method mentioned	https://www.senecapolytechnic.ca/programs/bycredential/microcredential.html
Dec. 30, 2024	York University		1) "micro-credential"	1) 1840 (first 50 screened)	No	Not focused on HPE	https://www.yorku.ca/science/programs/micro-credentials/
Dec. 30, 2024	Oshki-Wenjack		No search function available	N/A	No	No assessment method mentioned	https://www.oshki.ca/programs/indigenous-birthworker-postpartum-micro-credential-course/
Dec. 30, 2024	Sault College		No search function available	N/A	Yes. 37 in health category (most outline assessment methods		https://www.saultcollege.ca/micro-credentials<br><br>https://training.saultcollege.ca/collections/healthcare?page=5
May. 2, 2025	Conestoga		1) "micro-credential"	1) 848 results (first 50 screened)	No	No assessment method mentioned	https://continuing-education.conestogac.on.ca/micro-credentials?keywords=&selectedAreaOfInterests=Health+%26+Life+Sciences
May. 2, 2025	Humber		1) "micro-credential"	1) 441 results (first 50 screened)	No	No assessment method mentioned	https://humber.ca/continuous-professional-learning/learner-programs/micro-credentials.html
May. 2, 2025	Northern college	https://www.northerncollege.ca/?s=%22micro-credential%	1) "micro-credential"	1) 10 results	No	No assessment method mentioned	https://www.northerncollege.ca/?s=%22micro-credential%22

		22					
May. 2, 2025	Brock		1) "micro-credential"	1) 216 results (first 50 screened)	No	Nothing health	https://brocku.ca/continuing-education/learn/micro-credentials/
May. 2, 2025	Lambton College		1) "micro-credential"	1) 2	No	Nothing health	
May. 2, 2025	Cambrian College		1) "micro-credential"	1) 8	No	No assessment method mentioned	https://catalog.cambriancollege.ca/ADMC/#programdeliverytext
May. 2, 2025	University of Guelph		1) "micro-credential"	1) 46	No	No assessment method mentioned	
May. 2, 2025	Fanshawe		1) "micro-credential"	1) 175 (first 50 screened)	No	No assessment method mentioned	https://www.fanshawec.ca/programs-and-courses/programs-type/part-time-studies/micro-credentials
May. 2, 2025	University of Windsor		1) "micro-credential"	1)478 (first 50 screened)	Yes (1)		https://continue.uwindsor.ca/700/courses#Health%20Sciences
**Known websites with Microcredentials**							
Dec. 30, 2024	Michener Institute				Yes. 9 courses		https://michener.ca/continuing-education/ce-courses/micro-credentials-offered/
